# Characteristics of autism spectrum disorders in a sample of egyptian and saudi patients: transcultural cross sectional study

**DOI:** 10.1186/1753-2000-5-34

**Published:** 2011-11-03

**Authors:** Hanan Hussein, Ghada RA Taha, Afrah Almanasef

**Affiliations:** 1 Okasha's Institute of Psychiatry, WHO Collaborative Center for Training and Research, Ain Shams University Hospitals, Abbasia, Cairo, Egypt; 2 Al-Amal Complex for Mental Health, Dammam, Kingdom of Saudi Arabia

**Keywords:** autism, culture, illness behavior, developmental delay

## Abstract

**Background:**

Autism is a biological disorder with clearly defined phenomenology. Studies from the Middle East on this topic have been particularly rare. Little is known about the influence of culture on clinical features, presentations and management of autism. The current study was done to compare characteristics of autism in two groups of Egyptian as well as Saudi children.

**Methods:**

The sample included 48 children with Autism Spectrum Disorder. They were recruited from the Okasha Institute of Psychiatry, Ain Shams University, Cairo, Egypt and Al-Amal Complex for Mental Health, Dammam, Kingdom of Saudi Arabia. They were grouped into an Egyptian group (n = 20) and a Saudi group (n = 28). They were assessed both clinically and psychometrically using the GARS, the Vineland adaptive behavioral scale, and the Stanford Binnet IQ test.

**Results:**

Typical autism was more prevalent than atypical autism in both groups. There were no statistically significant differences in clinical variables like regression, hyperactivity, epilepsy or mental retardation. Delayed language development was significantly higher in the Egyptian group while delay in all developmental milestones was more significant in the Saudi group. The Vineland communication subscale showed more significant severe and profound communication defects in the Saudi group while the Gilliam developmental subscale showed significantly more average scores in the Egyptian group. Both groups differed significantly such that the age of noticing abnormality was younger in the Saudi group. The age at diagnosis and at the commencement of intervention was lower in the Egyptian group. The Saudi group showed a higher percentage of missing examinations, older birth order and significantly higher preference to drug treatment, while the Egyptian group showed a high preference to behavioral and phoniatric therapies, higher paternal and maternal education, higher employment among parents and higher family concern.

**Conclusion:**

Cultural context may significantly influence the age of noticing abnormality, the age of starting intervention, developmental and perinatal problems, family concerns about managing the problem as well as familial tendency for neurodevelopmental disorders, all of which have important impact on clinical symptomatology and severity of autism. Culture also influences significantly the ways of investigating and treating autism.

## Background

Autism spectrum disorders (ASDs) are complex neurodevelopmental disorders characterized by qualitative impairments in three domains: social interaction, communication, and repetitive, stereotyped behaviour. ASDs can have a detrimental impact on the well-being of affected individuals [1]. These symptoms often begin by the age of three years, and persist throughout the life span. ASDs are associated with mental retardation and seizure disorders in a significant number of cases, and are influenced heavily by genetic factors [2].

Studies from the Middle East on this topic have been particularly rare. In a survey on mental health research in the Arab world over a 25 years period, publications on child psychiatry, in particular, on topics such as autism, were found to be under-represented [3].

Although autism occurs in all cultures and countries, most of the published researches have come from Western countries. In particular, relatively little is known about its clinical correlates and comorbidity in Middle Eastern and Arab countries [4]. Recently, there has been some developing research in the area of autism in the Middle East. For example in Saudi Arabia, one study investigated 49 patients with autism, and found that; females were older than males at the time of referral, 11 patients had a history of seizure disorder, 25 patients were taking psychotropic medications and 14 patients were the product of consanguineous marriages [5].

In a study from the United Arab of Emirates (UAE), a representative random sample of 694 three-year-old United Arab Emirates national children was evaluated in a two-stage study in the community. In the first stage, using an autism screening questionnaire, 58 per 10,000 children were noted to have autistic features. In the second stage using a clinical interview, the weighted prevalence was estimated to be 29 per 10,000 for a DSM-IV diagnosis of pervasive developmental disorder (PDD). The presence of autistic features was associated with male gender, the presence of behavioral problems and a family history of developmental delay [6].

Recently, a study conducted in nine Arabic speaking countries, showed a significant increase of maternal health problems during pregnancy and labor for ASD mothers [7]. In addition, child health problems were more evident among ASD subjects as reported by their parents with significant differences from controls. A major strength of the study was that it was the first known study where Arab countries undertook a collaborative mental health investigation using the same tools for the screening of a specific disorder [7].

A recent study about ASDs in Arab Countries recruited a total of 37 boys and 23 girls from three Arab countries (Egypt, Saudi Arabia, Jordan) and found that the boys had poor emotional responsiveness and the girls had more cognitive problems [8]. Also the boys exhibited significantly more delinquent behavior problems. In another study that was conducted to determine the possible risk factors of autism in 100 patients with autism recruited from a pediatric hospital at Ain Shams University, 46% of patients presented at the age of one and a half years and 32% at the age of 2 years [9]. Moreover, 55% percent of patients had mild to severe retardation. High maternal age (mother, ≥ 35 years) at birth was found in 23% of autistic children. Also advanced paternal age (father, ≥ 35 years) at birth was found in 91% of cases. Positive family history was found to be significantly associated with the risk of autism (16% of cases versus 1% of controls). Also, postnatal factors such as history of hypoxia, resuscitation and history of jaundice were considered significant risk factors for autism [9].

To the authors' knowledge, there are few studies that compared established cases of autism in Arab countries simultaneously. The present study is one of the few clinical studies describing and comparing two samples of autistic children in two large Arab countries; Egypt and Saudi Arabia, simultaneously. Both are strategically important countries in the Middle East which face enormous problems of access to health care with under-developed services for children with developmental and psychiatric disorders [10].

### Hypothesis

Autism is a biological disorder with a clearly defined phenomenology. However, cultural differences might shape its clinical presentation as well as the way autistic children are dealt with generally and respect to clinical interventions.

### Objectives

This study was done to explore the condition of autism in two groups, one of Egyptian and one of Saudi children. It aimed at understanding and comparing the demographic background, clinical characteristics and presentations of autism as well as comparing methods of examination and intervention with this condition in both countries.

## Subjects and Methods

### Design and site of the study

This study was a cross-sectional study. Subjects were recruited over a period of 4 months from two centers with large catchment areas; from the Institute of Psychiatry, Ain Shams University Hospitals in Cairo, Egypt and from Al-Amal Complex for Mental Health in Dammam, Kingdom of Saudi Arabia. Inclusion criteria included children with Autism Spectrum Disorders of both sexes and with age ranging from birth up to 18 years. Comorbidity such as mental retardation and/or epilepsy was also included. Exclusion criteria were Rett's disorder or childhood disintegrative disorder as they were very rare. The authors recruited all cases who fulfilled the inclusion criteria during a three month period and gave informed consent through their legal guardians. The study was approved by the scientific and ethical committees of the Institute of Psychiatry, Ain Shams University and Al-Amal Complex for Mental Health.

### Procedure and Tools

After considering the inclusion and exclusion criteria, 20 Egyptian and 28 Saudi autistic children, diagnosed according to DSM-IV, were recruited [11]. A comprehensive approach to the assessment of autistic children was done including: 1) A clinical assessment sheet for symptoms of autism and associated symptoms such as hyperactivity, regression, seizures, and comorbid psychiatric conditions. 2) An assessment sheet for family factors (parents' education and work, patient education, family history of related disorders and family concern for autism). 3) An assessment sheet for perinatal events, birth order and developmental factors. 4) Clinical sheets were designed by the authors according to their knowledge of the literature and the DSM-IV symptom checklist for autism. 5) A sheet for detailed intervention and management (examinations, modality of treatment; drug, behavioral therapy and others). These sheets were applied directly by two child psychiatrists during a direct interview with patients and their caregivers. 6) An assessment of the severity of autism using the Gilliam autism rating scale (GARS) Arabic version: This test was used for diagnosis and assessment of the severity of autistic features for ages 3-22 years [12]. It consists of 56 items, subdivided into 4 subscales: communication, social interaction, stereotyped behaviors, development and total score. The Arabic version has been validated with good reliability and validity and used in many studies before [13]. The lower the scores are, the worse the condition is. 7) The Arabic version of Stanford Binet test fifth edition for assessment of intelligence [14]. This test was translated and standardized for use in Arab countries several years ago with good reliability and validity [15]. 8) The Vineland Adaptive Behavioral Scale (VABS) was used to assess the adaptive functions of patients [16]. The test includes four subdomains (communication, social skills, daily living, and motor skills) and a composite adaptive behavioral score. The higher the scores of this test, the better the adaptive functioning. The Arabic version was validated with good reliability and validity and used in many studies in Arab Countries [17]. The last three assessments were administered by two well trained and experienced clinical psychologists (an Egyptian psychologist for Egyptian group and another Saudi psychologist for the Saudi group). Parents were interviewed and the children were examined clinically first by the psychiatrists and then were referred to clinical psychologists within the following days for application of the GARS, VABS and Stanford-Binet test. Clinical psychologists were blind to the purpose of the study.

In preparation for this study all authors and the clinical psychologists met in a pilot study to test the interrater reliability and it was Κ = 0.72 for the psychologists and K = 0.74 for the psychiatrists.

Recognition of illness was assessed through asking about the age of noticing the abnormality. Reaction to illness was measured as age of starting intervention, regularity of follow up, missed examinations and types of interventions preferred. Family concern was measured through assessment for the regularity of follow up and proper response to requests and examinations.

### Statistical analysis

Data obtained was analyzed by an expert statistician using the Statistical Package of Social Sciences (SPSS) version 17. The statistician chose the best tests for small sample sizes. Numerical data were represented in the form of means and standard deviations. They were tested for normality using the Komogorov-Smirnov test. This non-parametric test was used because age was not evenly distributed which affected the normality of the sample. Normal data were compared using independent sample t tests (t) while non-normal data were compared using the Mann-Whitney (U) or Wilcoxon (W) tests. Categorical data were presented in numbers and frequencies and were tested for statistical associations using Chi square tests. Correlations were done using bivariate analysis.

## Results

### Sociodemographic characteristics of patients of both groups

Forty eight patients were included in the study. Subjects were grouped into 2 two groups; an Egyptian group (n = 20) and a Saudi group (n = 28). Both groups were matched regarding age and gender (table 1). However, the age at noticing abnormality differed significantly between Egyptian and Saudi patients, being earlier in Saudi patients. Conversely, age of diagnosis and starting intervention varied to a very highly significant degree, being lower in the Egyptian group. In the Saudi group, patients were significantly older in birth order when compared to the Egyptian group (table 1).

**Table 1:** Comparison between Egyptian and Saudi Groups in sociodemographic variables

Items	Egyptian group		Saudi group		X^2^	df	P value
					
	n	%	n	%			
Gender							
Male	12	60%	18	64.3%	0.091	1	0.4
					
Female	8	40%	10	35.7%			

	**mean**	**SD**	**Mean**	**SD**	**U**	**Z**	**P value**

Age in years							
Total sample	7.4	4.02	7.6	3.4	253.5	-0.5	0.5
					
Male	8.2	4.2	7.8	3.6			
					
Female	6.1	3.5	7.3	2.9			

Age of noticing abnormality							
Total sample	2.25	0.5	1.7	1.3	188	-1.9	0.04
					
Male	2.4	0.5	1.5	1.2			
					
Female	2	0.5	2.1	1.3			

Age of starting intervention							
Total sample	2.6	0.9	4.8	2.5	136.5	-3.07	0.002
					
Male	3.1	0.4	4.8	2.8			
					
Female	3	1.5	4.7	1.9			

Birth order	1.4	0.8	2.9	2.4	150.5	-2.8	0.004

### Clinical characteristics of patients of Egyptian and Saudi groups

There was no difference of statistical significance between the two groups in type of autism. Also, no significant differences were found between both groups regarding presence of seizures, hyperactivity, history of regression, comorbid psychiatric problems and positive findings in examination (table 2). On comparing male and female patients of the whole sample, hyperactivity was statistically significantly associated with male gender (male: 21(70%) vs. female: 6 (33.3%), X^2 ^= 6.1, df = 1, P value = 0.01)

**Table 2:** Clinical data in both groups

Items		Egyptian	Saudi	X^2^	df	P value
					
		n (%)	n (%)			
Diagnosis	Autism	19(95%)	23 (82.1%)	1.76	1	0.1
	PDDNOS	1 (5%)	5 (17.9%)			

Regression	Yes	6 (30%)	6 (21.4%)	0.46	1	0.49
				
	No	14 (70%)	22 (78.6%)			

Hyperactivity	Yes	10(50%)	17 (60.7%)	0.54	1	0.46
				
	No	10 (50%)	11 (39.3%)			

Epilepsy	Yes	1 (5%)	7 (25%)	3.36	1	0.07
				
	No	19 (95%)	21 (75%)			

Psychiatric Comorbidity	Yes	5 (71.4%)	19 (67.9%)	0.03	1	0.6
				
	No	2 (28.6%)	9 (32.1%)			

Clinical examination	Positive physical signs	2 (10%)	1 (3.6%)	4.8	3	0.1
				
	Positive behavioral signs	6 (30%)	16 (57.1%)			
				
	Positive physical & behavioral	5 (25%)	7 (25%)			
				
	Normal	7 (35%)	4 (14.3%)			

### Psychometric characteristics of patients of both groups (severity)

IQ scores, Vineland ABS and Gilliam scales were compared between both groups using t-tests and Mann-Whitney tests. There were significant differences in the stereotype and developmental Gilliam subscales. Saudi children showed significantly more stereotype and lower developmental abilities in the Gilliam scores than the Egyptian group. There was no statistical significant difference between both groups regarding level of intelligence (table 3).

**Table 3:** Comparison between both groups regarding psychometric assessments

Items	Egyptian Mean, SD	Saudi Mean, SD	t(U)	df (Z)	p Value
IQ	65.5 ± 20.9	63.1 ± 23.1	(259)	(-0.4)	0.6

Guilliam scale (total)	93.8 ± 14.7	86.5 ± 14.6	1.69	46	0.098
	
1. stereotype	7.5 ± 3.3	9.57 ± 3.1	(182)	(-2.06)	0.04
	
2. communication	6.26 ± 2.8	8.03 ± 3.1	(146.5)	(-0.23)	0.8
	
3. social interaction	8.75 ± 3.3	6.85 ± 3.32	(197)	(-1.7)	0.08
	
4. developmental	10.7 ± 1.7	8.6 ± 3.3	(142)	(-2.16)	0.03

Vineland ABS	44.5 ± 15.16	41.75 ± 19.8	0.69	45	0.49
	
1. Communication	39.4 ± 13.7	41.3 ± 18.9	(221.5)	(-0.96)	0.3
	
2. Daily skills	46.4 ± 17.2	41.6 ± 23.5	(226.5)	(-0.86)	0.38
	
3. Socialization	48.2 ± 17.6	47.4 ± 21.03	0.13	45	0.89
	
4. Motor	62.4 ± 18.9	66.15 ± 20.8	-0.45	22	0.65

The Vineland communication subscale showed significantly more severe and profound communication defects in the Saudi group whereas the mild and moderate communication defects were more common in the Egyptian group. The Gilliam developmental subscales showed significantly more average scores in the Egyptian group, while there were more low, very low and below average scores in the Saudi group (table 4). On comparing males and females of the whole sample, female patients showed more statistically significant above average and average ratings on the total Gilliam scores than males (female: 12 (66.7%) vs. male: 14 (46.6%), X^2 ^= 9.1, df = 3, p = 0.02)

**Table 4:** Severity of autistic symptoms in both groups

Scales		Egyptian	Saudi	X^2^	df	P value
					
		n (%)	n (%)			
Vineland communication subscale	Profound	0	4 (14.3%)	9.8	4	0.04
				
	Severe	4 (21.1%)	10 (35.7%)			
				
	Moderate	7 (36.8%)	3 (10.7%)			
				
	Mild	8 (42.1%)	8 (28.6%)			
				
	Moderately low	0	3 (10.7%)			

Gilliam developmental subscale	Average	17 (85%)	8 (34.8)	12.2	4	0.01
				
	Below average	1 (5%)	7 (30.4%)			
				
	Low	0	3 (13%)			
				
	Very low	0	1 (4.3%)			
				
	Above average	2 (10%)	4 (17.4%)			

### Familial and perinatal background of Egyptian and Saudi autistic patients

Family concern was significantly higher in the Egyptian group (table 5). Delayed language development was also significantly higher in the Egyptian autistic children, while delay in all developmental milestones was more significant in the Saudi autistic children. Also, the Saudi group showed more significant abnormal family history in terms of more consanguinity, ASDs, delayed language development and mental retardation when compared to the Egyptian group.

**Table 5:** Family history and concern, perinatal and developmental problems in both groups

Items		Egyptian	Saudi	X^2^	df	P value
					
		n (%)	n (%)			
Family concern	Concerned	19 (95%)	15 (53.6%)	9.7	1	0.002
				
	Not concerned	1 (5%)	13 (46.4%)			

Family history	Consanguinity	1 (5%)	5 (17.9%)	25.5	4	0.000
				
	Autism	3 (15%)	7 (25%)			
				
	DLD	2 (10%)	11 (39.3%)			
				
	MR	1 (5%)	5 (17.9%)			
				
	Negative	13 (65%)	0			

Perinatal problems	Yes	7(35%)	12 (42.9%)	0.3	1	0.76
				
	No	13 (65%)	16 (57.1%)			

Developmental history	Delayed Milestones	1 (5%)	14 (50%)	17.2	2	0.000
				
	DLD	19 (95%)	10 (35.7%)			
				
	Negative	0	4 (14.3%)			

DLD: Delayed Language Development MR: mental retardation

High paternal and maternal education and high employment among parents of autistic children were significantly more preponderant in the Egyptian group (table 6). Also, a high percentage of the Egyptian autistic children were in private schools while the majority of Saudi patients were in governmental schools. This is accounted for by better governmental educational services for autistic children in Saudi Arabia and paucity of educational services for autistic children in Egypt.

**Table 6:** Education and work profile of patients and their parents in both groups

Items		Egyptian n (%)	Saudi n (%)	X^2^	df	P value
Paternal education	high	19 (95%)	6 (21.4%)	25.4	2	0.000
				
	middle	1 (5%)	12 (42.9%)			
				
	low	0	10 (35.7%)			

Maternal education	high	16 (80%)	9 (32.1%)	11.5	2	0.003
				
	middle	3 (15%)	8 (28.6%)			
				
	low	1 (5%)	11 (39.3%)			

Patient education	Governmental school	0	12 (42.9%)	21.2	3	0.000
				
	Private school	11 (55%)	9 (32.1%)			
				
	Special needs	7 (35%)	0			
				
	None	2 (10%)	7 (25%)			

Father work	Employed	9 (45%)	16 (57.1%)	4.85	3	0.1
				
	Skilled	1 (5%)	2 (7.1%)			
				
	Other	10 (50%)	7 (25%)			
				
	Unemployed	0	3 (10.7%)			

Mother work	Employed	8 (40%)	2 (7.1%)	6.5	2	0.01
				
	Skilled	0	2 (7.1%)			
				
	Unemployed	12 (60%)	24 (85.7%)			

### Management of autism in both groups (investigations and treatment modalities)

Data were gathered regarding the examinations and treatment modalities tried since diagnosis. Data of both groups was compared using chi square tests and results are shown in the following table (7).

The Egyptian group showed significantly more normal results in audiometric and radiological examinations in comparison to the Saudi group. Also, the Saudi group showed a higher percentage of missed examinations (table 7) than the Egyptian group. Although combined behavioral and drug therapy is the most common intervention among both groups (> 50%), 42.9% of Saudi patients showed significantly higher preference for drug treatment only, of which about 71.4% were stable on mono/polytherapy. On the other hand, the Egyptian group showed a significantly higher preference for behavioral and phoniatric therapies.

**Table 7:** Investigations and treatment modalities practiced among Egyptian and Saudi groups

Examinations and interventions		Egyptian n (%)	Saudi n (%)	X^2^	df	P value
Radiology	Normal	15 (75%)	6 (21.4%)	7.93	2	0.000
				
	Abnormal	3 (15%)	2 (7.1%)			
				
	Not available	2 (10%)	20 (71.4%)			

Audiometry	Normal	19 (95%)	14 (50%)	11.03	2	0.004
				
	Abnormal	0	2 (7.1%)			
				
	Not available	1 (5%)	12(42.9%)			

Type of intervention	Drugs only	2 (10%)	12 (42.9%)	13.5	3	0.004
				
	Behavioral only	6 (30%)	0			
				
	Combined	10 (50%)	15 (53.6%)			
				
	Others	2 (10%)	1 (3.6%)			

Drug treatment	No drug	8 (40%)	0	13.5	3	0.004
				
	Stable monotherapy	6 (30%)	14 (50%)			
				
	Stable polytherapy	3 (15%)	6 (21.4%)			
				
	Changeable drugs	3 (15%)	8 (28.6%)			

Behavioral treatment	Yes	17 (85%)	15 (53.6%)	10.1	1	0.006
				
	No	3 (15%)	13 (46.4%)			

Phoniatric treatment	Yes	18 (90%)	7 (25%)	19.7	1	0.000
				
	No	2 (10%)	21 (75%)			

## Discussion

Culture is defined as the characteristic ways in which people of certain group perceive and interact with their environment. Moreover, it is the external expression of people's mental life in the form of language, beliefs, customs, technology, human relationship, and many other factors [18]. Illness behavior is the way that mental illness is recognized, labeled, explained and treated within any particular culture [19].

Although Egypt and Saudi Arabia belong to Arab Islamic developing countries, they differ in their values, beliefs, customs, social relationships and economic burdens.

The culture of Egypt has six thousand years of recorded history. For millennia, Egypt maintained a strikingly complex and stable culture that influenced later cultures of Europe, the Middle East and Africa. After the Pharaonic era, Egypt itself came under the influence of Hellenism, for a time Christianity, and later, Islamic culture [20]. Egypt is a low-income developing country. The major provider of care is the Ministry of Health, which runs a nationwide system of health services. MOH services are subsidized, and provided largely free to all citizens. The Education Ministry through its budget supports twenty university hospitals. These provide a higher quality of care than MOH facilities. While public provision dominates inpatient care services, Egyptians make considerable use of private services. Private clinics and hospitals are staffed for the most part by government doctors. These private services are all funded by private out-of-pocket spending [21]. Services for children with autism are offered primarily in private clinics and hospitals in addition to university hospitals. Mendoza and his colleagues tried to find the economic costs of ASD in Egypt, and compared these costs with those of developed countries [22]. They discovered that care and support for ASD are typically based on a household-provider model, in contrast to western, institution-based models. ASD costs in Egypt largely derived from much higher investments in time, effort and behavioral adaptation on the part of family caregivers.

The cultural setting of Saudi Arabia is a restrictive Muslim culture. Traditional values and cultural mores are adapted into legal prohibitions. Alcoholic beverages are prohibited as are pork products. Popular forms of media entertainment are banned or permitted under tight controls to prohibit the spread of immoral words, images or ideas [23]. The Ministry of Health is responsible for the supervision of healthcare and hospitals in both the public and private sectors. The healthcare system has a network of primary healthcare centers and clinics that provide preventive, prenatal, and basic services [24]. Some of mental health hospitals have free child psychiatry clinics and some rehabilitation services but the majority of hospitals have not. There are private services of rehabilitation for children of ASDs and supported from the ministry of social affairs but only accessible in large cities [25]. That is the reason why, compared to Egypt, care and support for children with ASDs in Saudi Arabia are largely derived from institution based models with much lower investments in time, effort and behavioral adaptation on the part of family caregivers.

Studies indicate that parents' perceptions of the nature of a disability may differ to some degree, based on their cultural values [26,27]. In many Arab groups, violating a religious code is believed to be a cause of disability, especially when rational explanations of disability are not clear [27]. The child's disability tended to produce feelings of shame and guilt among Arab societies [28]. Parental perceptions about the causes of disability have a tremendous impact on parents' behaviors in terms of seeking help or intervention for their children or the kind of help they look for, and their support of the treatment process. Ryan and Smith report that disagreements may exist between the parents' beliefs about physical, supernatural, and metaphysical causes of disability, and the Western diagnoses and professionals' beliefs [29]. This conflict may lead the parents to seek some alternative cures like sociocultural, folk, or religious remedies [30]. Studies also reveal that even parents from the same cultural backgrounds may hold different beliefs, based on their level of acculturation, socioeconomic status, and education [31,32]. An Arab study tried to explore the extent to which general education teachers accept the inclusion of students with disabilities in mainstream classrooms [33]. The results indicated that the overall attitudes of educators towards persons with disabilities were negative. Moreover, the study concluded that there is more work to be done on the development of an 'inclusion culture' among teachers [33].

While exact figures are not available, anecdotal reports suggest an increase in the prevalence of autism in both countries. In the current study, we investigated the clinical, familial, developmental, and interventional profiles in both countries. Moreover, we investigated how the culture shaped some dimensions of the illness behavior as symptom recognition and response to illness.

### Sociodemographic characteristics of patients

Autism is commonly reported in literature to have higher incidence in males than females. Fernell and colleagues reported a ratio of 5.5:1 in Sweden [34]. Others reported sex ratio of 3:1 [5,35]. In the current study, the male to female ratio among the whole sample was 1.6:1, being nearly equal in both groups (1.5:1 in Egyptian and 1.8:1 in Saudi group) which is less than that reported in other studies [5,35]. Approximately the same ratio (1.6:1) was also found in another study on a sample of patients from Egypt, Saudi Arabia and Jordan in which the number of boys was 37 and the girls 23 [8]. These results should be taken with caution as the sample in the current study is not a community representative sample neither with respect to sample size nor methodology of recruiting patients, thus cannot be granted high value for discussing sex ratio. It might only indicate that families of patients are nearly equally concerned with affected male and female offspring and not essentially with males. Within the Saudi sample, however, the age of noticing abnormality or recognition of illness was significantly earlier in Saudi males than Saudi females (1.5 ± 1.2, 2.1 ± 1.3 respectively) (table 1). This is in accordance with Al-Salehi and colleagues who found that Saudi females were significantly older at the time of the referral (males, mean age 5.8 ± 3.2; females, mean age 7.8 ± 3.1; unpaired t test; p < 0.02) [5]. It might be that female patients in this sample showed less severe symptoms than male patients which could have led to a delay in noticing abnormality. Another explanation might be masculine cultural influence which is still especially evident in Saudi society [36]. Meanwhile, the age of noticing abnormality was almost equal in both Egyptian males and females (table 1) which was similar to an Indian study reporting no difference found for age of autism recognition based on the sex of the child in the Indian context [37].

Another important finding was that the patients were significantly older in terms of birth order in the Saudi group than in the Egyptian group (table 1). Observationally, Saudi culture is characterized by younger age of marriage among males and females as well as higher birth rate which is no longer the case except in rural Egyptian culture. Due to better educational background, the stoppage rule may be acting more in the Egyptian group. The importance of birth order was also emphasized in the study of Juneja and colleagues who reported that the age of presentation was significantly earlier in firstborn children (2.28 years) as compared to later-born children (3.6 years) [38]. This observed difference might be attributable to parents spending more time with first-born children.

One of the really striking results was the age of noticing abnormality which was significantly earlier in Saudi patients when compared to Egyptian patients. The age of starting intervention and seeking medical help was the reverse i.e. significantly earlier in Egyptian than in Saudi patients (table 1). As for the Egyptian group, age of diagnosis and start of intervention was even younger (2.5 ± 0.9) than in western countries where the median age of diagnosis for autism decreased from 4 years to 3 years of age throughout the 1990s - 2000s [39]. While for Saudi group, it is still above 4 years. This was similar to findings of Tang and colleagues who reported that the majority (93%) of autistic children in Hong Kong were referred before the age of 6 years [35].

Overall, the difference between age of noticing abnormality and age of diagnosis and intervention was minimal in the Egyptian group but in the Saudi group, it was a marked difference (about 2 years).

It can be inferred that the age of recognition of symptoms is an indicator for the knowledge of parents about the illness and their level of denial. Hence, the younger age of noticing the symptoms in the Saudi group may indicate more knowledge and less denial in Saudi culture than in Egyptian culture. It is well known that the level of knowledge about autism in Saudi culture is good due to the efforts of many nongovernmental organizations [5]. Moreover, Egyptians are characterized by their warm emotions and their overprotective attitude towards their children which is why the level of denial may be higher in Egyptian culture [40].

In contrast, the age of starting intervention was significantly earlier in Egyptian than in Saudi patients (table 1). This difference between groups may have several reasons, including the significant differences in parents' education and family concern between the two cultures (tables 5, 6). The higher paternal and maternal education and higher employment among parents of autistic children in the Egyptian group may explain higher concern among the Egyptian parents and thus consultation for early treatment interventions. Moreover, earlier intervention in Egyptian group may reflect easier access to services in Egypt. In Saudi Arabia, delayed intervention may also indicate limited accessibility to services and more tolerance by the extended families.

### Perinatal, developmental and family abnormalities

Abnormal family history was significantly more apparent in the Saudi group, represented in higher incidence of family history of consanguinity and neurodevelopmental disorders such as autism, delayed language development and mental retardation (table 5). In a similar Saudi study, Al-Salehi and colleagues reported 14 autistic subjects (28.57%) with a history of consanguinity [5]. There is a well established constant observation between higher prevalence of all genetically transmitted disorders and consanguineous marriages [41]. Consanguinity is more evident in Saudi culture [42]. The consanguinity rate is in excess of 50% and is a practice that remains strongly embedded within Saudi culture [43]. In, 1995 El-Hazmi and colleagues reported that the prevalence of consanguinity in Saudi Arabia ranges from 34 to 80% depending on local circumstances [44].

Perinatal problems are also more prevalent among Saudi group, yet, this association was not of statistical significance. Moreover, the Saudi group showed more delay in all developmental milestones while the Egyptian group showed more delayed language development (table 6). This may explain the common presentation with delayed language previously reported in Egyptian autistic children which is in accordance with Tang and colleagues who found that the most common reason for referral was language delay (39%) [45,35]. The delay in all milestones in the Saudi group, reported in this study, may be related to consanguinity and/or perinatal complications and may further explain the younger age of noticing abnormality among Saudi group. Similar to findings in Saudi group, Juneja and colleagues reported 96% children in their study had developmental delay in all milestones, whereas 27.5% had significant perinatal events [38].

### Clinical characteristics of patients

Referring to the diagnosis, the number of patients with typical autism in both groups was significantly more than those with atypical autism (PDDNOS) which is consistent with the prevalence of autistic spectrum disorders in DSM-IV [11]. Similarly, Al-Salehi and colleagues reported that in a sample of 49 Saudi autistic patients, the most common diagnosis was autism (n = 44), followed by pervasive developmental disorders not otherwise specified (n = 5) [5].

Usually in western studies, the number of PDDNOS or atypical autism is much more than that of typical autistic disorder [46,47]. For example, in a study from UK there was a more marked increase for PDDs other than autism [47]. Cross cultural reasons may play a role in this finding. Other reasons may be the different sampling techniques used in different studies.

Results showed that 30% of Egyptian patients and 21.4% of Saudi patients had a history of regression. Also, 50% of Egyptian patients and 60.7% of Saudi patients showed hyperactivity. Epilepsy was present in 5% of the Egyptian group in comparison to 25% of Saudi group. Psychiatric comorbid problems were reported in 71.4% of Egyptian versus 67.5% of the Saudi group. However, both groups when compared together showed no statistical differences regarding the above symptomatology (table 2). Most studies from industrialized countries suggest that the prevalence rate of the regressive form of autism to be around 20% [48]. In a recent Swedish study, Fernell and colleagues tried to characterize the panorama of developmental disorders in 208 preschool children with a clinical diagnosis of autism spectrum disorder (ASD) and found that 22% of the total group experienced a period of regression, including speech and language [34]. Moreover, epilepsy had been diagnosed in 6% of the children. About 40% of the group exhibited hyperactivity [34]. The results of the Egyptian sample in the current study were similar to the Swedish study (30%, 5% & 50% for regression, epilepsy and hyperactivity respectively). The low percentage of epilepsy in the Egyptian group may be related to better developmental functioning. Comparing the current Saudi group to another previous Saudi study, Al-Salehi and colleagues reported hyperactivity and aggression in 44.8% of their Saudi patients; epilepsy was found in 22.4% as compared to 25% in our Saudi patients and almost half of them (n = 22) were referred for co-occurring behavioral problems, in particular, hyperactivity and aggression [5]. The higher incidence of epilepsy in the Saudi group may be attributed to higher genetic loading, more developmental and perinatal problems. We might even conclude that maintenance of follow up and seeking of services in Saudi Arabia is mainly due to the presence of epilepsy and behavioral problems rather than autism itself.

### Psychometric characteristics of both groups

On a psychometric level, both groups showed no statistical differences in the Vineland Adaptive Behavior scale scores (table 3). These findings are in accordance with Fenton and his colleagues who compared autistic patients to those with moderate to severe developmental delay and reported fairly homogeneous adaptive behavior profiles in both groups [49]. Specifically, when speaking in grades, Saudi group showed significantly more severe and profound communication defects in the Vineland communication subscale while more mild and moderate communication defects were found in the Egyptian group. This may explain the reporting of Al-Salehi and colleagues that communication deficits were the most common cause for referral of Saudi autistic children [5]. Perry and colleagues reported a characteristic "autism profile" whereby the Socialization and Communication scales were lower in autism [50]. Freeman and colleagues also reported that the rate of growth in Communication and Daily Living Skills was related to initial IQ while the rate of growth in Social Skills was not [51].

In the Gilliam scale, the Saudi group showed scores indicating significantly more stereotypes and developmental deficits in comparison to the Egyptian group (table 3). This finding can be attributed to more perinatal, developmental and consanguinity problems in the Saudi group rather than to autism itself. When considering severity in terms of grades, the Gilliam developmental subscale showed significantly more average scores in the Egyptian group while there were more low, very low and below average scores in the Saudi group (table 4). In the whole sample, being male was more correlated with hyperactivity and poorer total Gilliam scores. Previous studies were controversial on this issue. McLennan and colleagues reported males to be rated more severely autistic than females on several measures of early social development, but not in any other areas [52]. However, Volkmar and colleagues reported that sex differences were primarily confined to IQ, but were not prominent in other measures of severity of autism [53].

### Comparison between both groups regarding clinical practice

On the level of response to illness the Egyptian group showed more response to requests of examinations. Missed examinations were more frequent in the Saudi group which may be due to many causes: 1) limited access to services and long waiting lists in Saudi hospitals [5], 2) more dependence on governmental free services, 3) decreased awareness as regards importance of these examinations, and 4) lower educational levels in the Saudi culture [5].

Moreover, radiological and audiometric normal findings were more frequent in the Egyptian than in Saudi group (table 7). This difference may be related to a highly significant difference found in the presence of developmental delay in the Saudi group. Also it is related to the difference found in the presence of more perinatal complications in the Saudi group; however, the latter difference was not statistically significant.

On interventional levels, combined drug and behavioral therapies were the most common in both groups, yet, there was more preference towards behavioral than drug therapy within the Egyptian group. In contrast, the Saudi group showed more preference towards drug therapy rather than behavioral therapy. This may be attributed to cultural differences in acceptance of psychiatric drug therapy in children and to differences in dealing with autism as well. Additionally, the Saudi group showed higher stability on monotherapy while the Egyptian group showed more changeability in drug treatment. A Saudi study by Al-Salehi and colleagues reported that 25/49 subjects were taking psychotropic medications for the purpose of behavioral symptoms and a significant number of patients were on medications for the control of superadded symptoms such as hyperactivity and aggression [5]. Similarly, Oswald and Sonenklar reported 83% of autistics had at least one drug claim during one year [54]. While in a recent study investigating the patterns of psychotropic medication use among 5,181 children with autism in USA, Rosenberg and colleagues reported that 35% used at least one psychotropic medication, most commonly stimulants, neuroleptics, and/or antidepressants [55]. The majority of psychotropic medications were prescribed for older age, or in the presence of intellectual disability or psychiatric comorbidity, and when the patient resided in a poorer county [55]. Moreover, the Egyptian group showed significantly higher preference to phonetic therapy than the Saudi group. This may be related to the availability and quality of services provided in both countries, which is more developed in Egypt than in Saudi Arabia.

#### Strengths and limitations

The authors tried to control threats to the internal and external validity: 1) All cases who presented to the outpatient's clinics through the five working days of the week were recruited to the study 2) the tools included clinical as well as psychometric testing to describe the sample, 3) all the tools used were standardized and validated, not merely translated, 4) to avoid overestimation or underestimation of parameters of interest, clinical psychologists were blind to the purpose of the study, 5) statistical data analysis was done by an expert statistician who chose the appropriate tests relevant to study rationale, sample size and generalizability.

In Arab countries, many studies have already been conducted using the GARS or the CARS, both of which are already translated and tested for reliability and validity. The investigators of the current study found these tests to be largely appropriate. In our opinion, it is only the items relating to social and emotional reciprocity, and adaptation to change, which might be inappropriate, both of which did not vary to a great extent between the Egyptian and Saudi cultures. The results of the current study should be taken with caution as the sample is not a community representative sample neither with respect to sample size nor methodology of recruiting patients. This is the primary limitation of the current study.

## Conclusion

Autism is biological disorder. It exhibits the same core deficits in all cultures. However, the pattern and timing of its presentation differs from one culture to another. The cultural context may significantly influence the age of recognition of illness, the age of starting intervention, presence of developmental and perinatal problems, family concerns about managing the problem as well as familial tendency for neurodevelopmental disorders, all of which have important impact on clinical symptomatology and severity of autism. Culture also influence significantly the ways of examining and treating autism. These cultural effects will lead to early detection or delay in detection of autism, thus may affect the early intervention and outcome of autism.

## Recommendations

Dependence on studies from Western culture and subcultures may mislead service planners and professionals. It is important for Eastern and developing countries to have their own research and to increase public awareness and integrated services for the problem of autism. Moreover, making autism care and support available, affordable and reliable should be a major health concern.

Professional interactions with patients' families require being responsive to their individual cultural perceptions, expectations, and practices. The interaction between professionals and parents must include knowledge of and respect for families' cultural beliefs in order to find common ground to help the patients.

## Competing interests

Apart from the fact that the psychiatrist (GRAT) who collected the clinical data from the Saudi sample was Egyptian, the authors declare that they have no competing interests.

## Authors' contributions

Both GRAT and HH conceived of the study and participated in its design and coordination. HH administered the instruments and collected the data from Cairo. GRAT administered the instruments and collected the data from Dammam. AA was the clinical psychologist who collected the psychometric data from the Saudi sample. All authors directed and oversaw the statistical analysis, participated in the writing and revision and approved the final manuscript.
